# Supplementary figures and images for: Transmission dynamics and vaccination strategies for Crimean-Congo haemorrhagic fever virus in Afghanistan: A modelling study
Source: PLoS Negl Trop Dis. 2022 May 23;16(5):e0010454. doi: 10.1371/journal.pntd.0010454 (PMC9166359; doi:10.1371/journal.pntd.0010454)

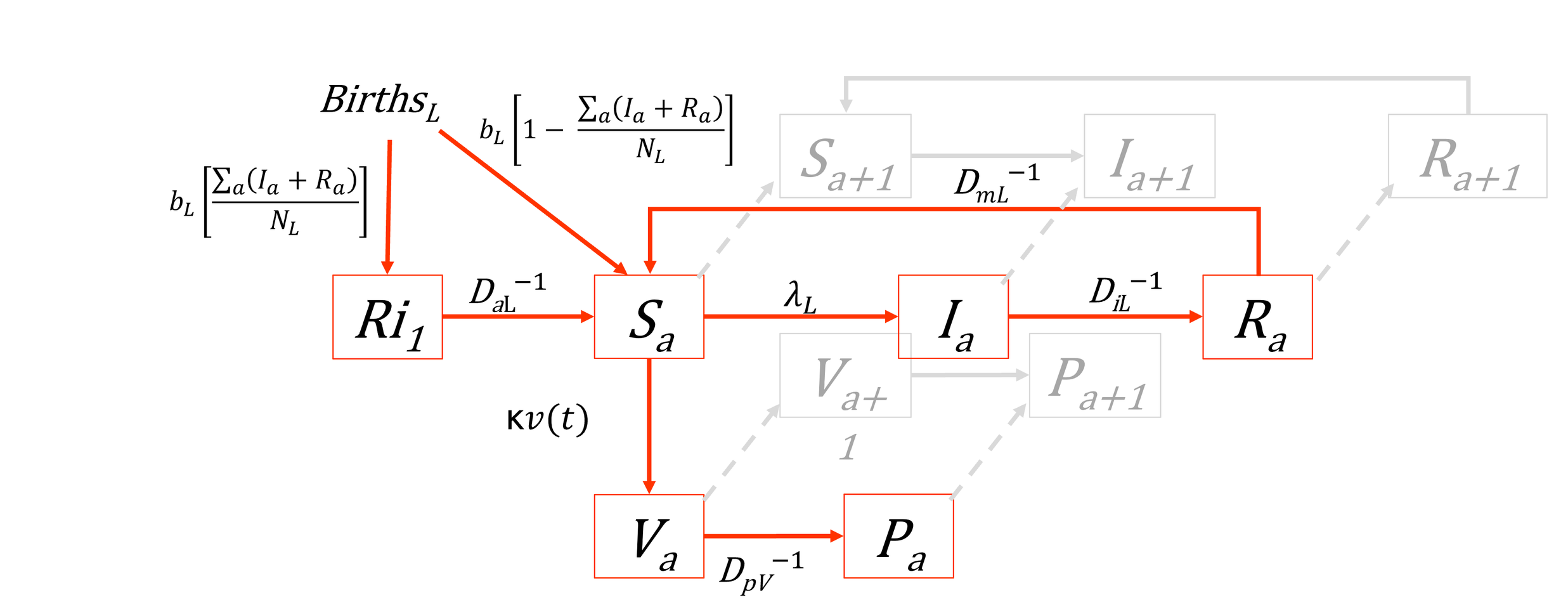

Supplement: S1 Fig — The structure shows the compartments and state transitions for livestock. Births occur at a rate equal to the mortality rate to maintain a population at equilibrium. Mortality rates are estimated to achieve a known livestock age-distributed population (see S2 Text). Offspring from prevalent CCHFV animals acquire transient immunity at birth through first colostrum (Ri). This immunity lasts for an assumed average period of 6 months. After this period, livestock move to the susceptible stage (S1). Susceptible livestock (Sa) acquire CCHFV with a force of infection λL that leads to an infectious period (Ia) with mean duration DiL, expressed as inverse time rate DiL-1. We assume that livestock lose immunity at a rate DmL.. Vaccination is implemented by recruiting susceptible animals at a rate ν(t). The effective number of immunised livestock is also defined by the efficacy of the vaccine (κ). At first, vaccination does not confer immunity Va, which is only acquired after a period of length DpV. In this structure, subscript a points to the age category within the age structure, over which transitions occurs as depicted with the shaded grey structure in the background (TIF) [file pntd.0010454.s001.tif]

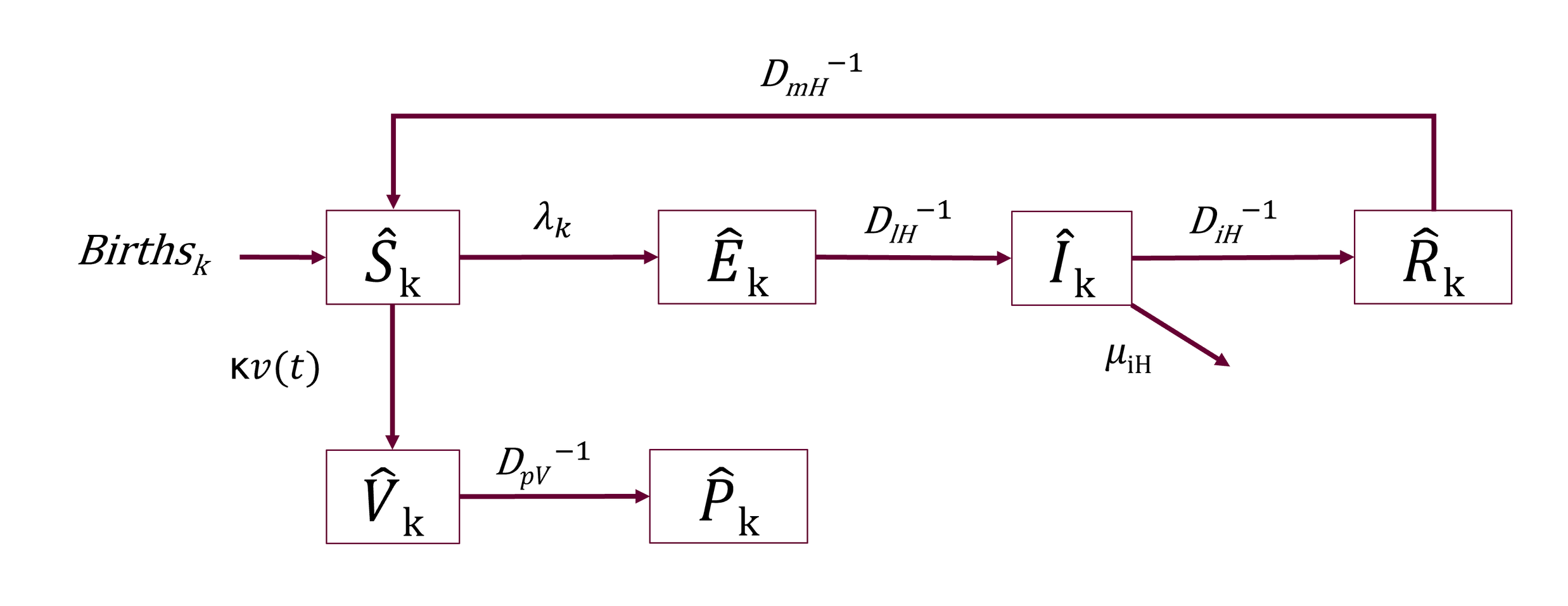

Supplement: S2 Fig — Humans are born at a rate reflecting the life expectancy in Afghanistan (keeping population size constant in the absence of infections), and split into the two human categories considered in this model. Namely farmers (the high-risk group) and other occupations. This distribution is taken from previous USAID surveys in the country (see parameters Table 1 in the main text). The categorisation by occupation in the model is reflected in this structure and in the mathematical equations using subscript k (0 = farmer; 1 = others). Infection is acquired in humans with force of infection λk, with differential risk k. Infection is followed by a latent period E^k with mean duration DlH that leads to an infectious period I^k. This infectious period can lead to either recovery R^k or death. Death from CCHFV in humans is described in the model as the competing hazard μiH that summarise the case fatality ratio for CCHFV. We assume waning immunity in humans that leads back to susceptible stage at a rate DmH-1. Vaccination occurs at rate v(t), differential by occupation. Effective number of immunised people is finally defined by the efficacy of the vaccine (κ). Vaccine protection occurs after vaccination after a mean period DpV. (TIF) [file pntd.0010454.s002.tif]

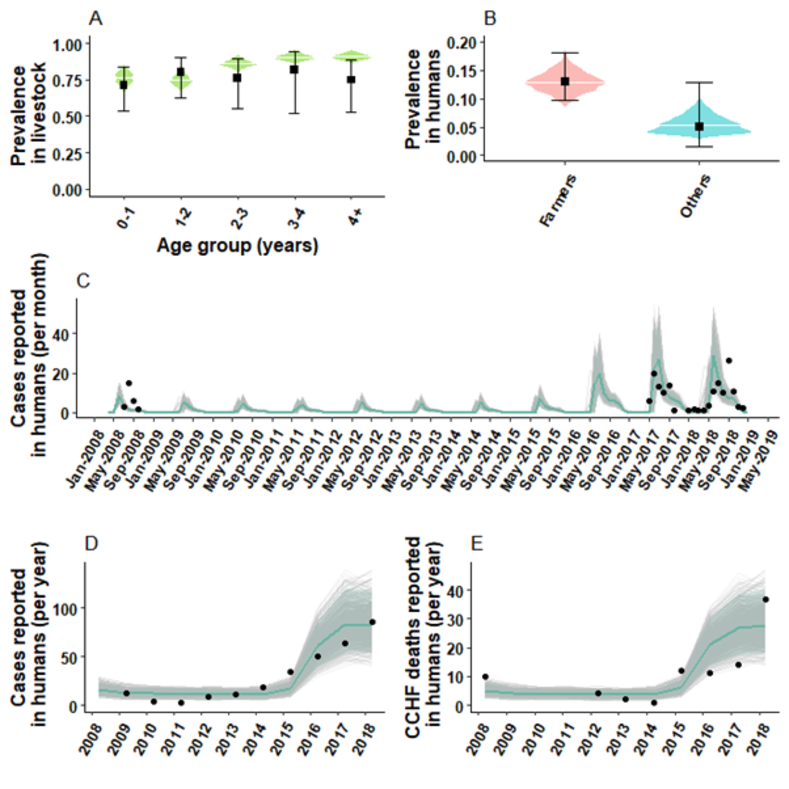

Supplement: S3 Fig — Panel A shows the age stratified simulated CCHFV IgG prevalence among livestock (green density plot), with the median estimate (white horizontal line), against IgG prevalence data for the same age groups as reported by Mustafa et al [14] from Herat (black square shows the mean and error bars the 95%CI). Panel B shows the posterior density and median estimate of IgG prevalence for the population of farmers and other occupations (density plots pink and blue) against IgG prevalence data from Herat reported. We take the prevalence estimate to match the dates of data collection as reported by Mustafa et al. Panel C shows stochastic model trajectories (grey lines) for monthly incident CCHFV human cases reported in Herat. In shaded pale grey, the 95% CrI and in solid blue, the median estimate. In black dots, monthly incident cases reported in two separate CCHF outbreaks in Herat: in 2008 as reported by Mofleh et al [16], and 2017–2018 as reported by Niazi et al, and Sahak et al [15,17]. In Panels D and E, yearly CCHF cases and deaths reported from Herat, against data (black) as reported by Sahak et al. (TIF) [file pntd.0010454.s003.tif]

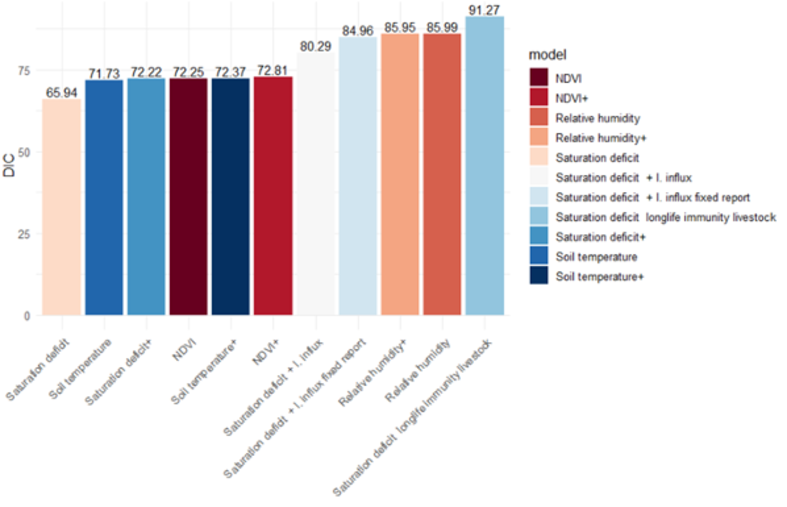

Supplement: S4 Fig — As mentioned in the main text, saturation deficit with baseline assumptions about reporting produces the lowest DIC (best model fit). Importantly, a model with lifelong immunity among livestock shows the worst performance. (TIF) [file pntd.0010454.s004.tif]

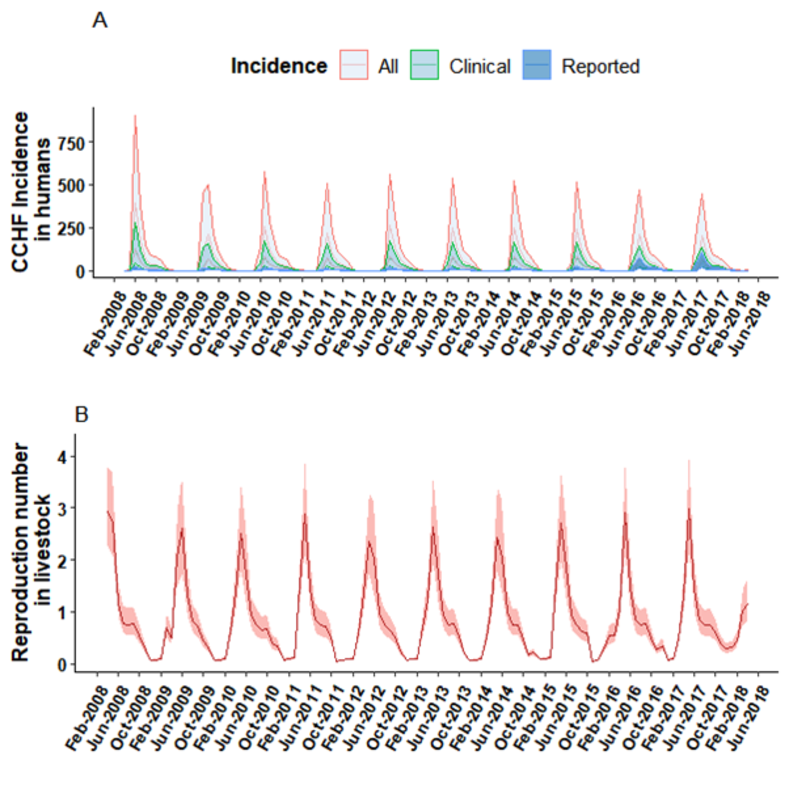

Supplement: S5 Fig — Transmission dynamics of CCHF in Herat, Afghanistan 2008–2018 In panel A, simulated trajectories of monthly CCHF incidence in a spectrum from reported clinical cases (blue shade), to all clinical cases (green) and all cases (red) including symptomatic/subclinical cases. The shaded area shows the 95% CrI. In Panel B, the simulated effective reproduction number for CCHFV in livestock. These are results for the final selected model, i.e., “saturation deficit driver” model. (TIF) [file pntd.0010454.s005.tif]

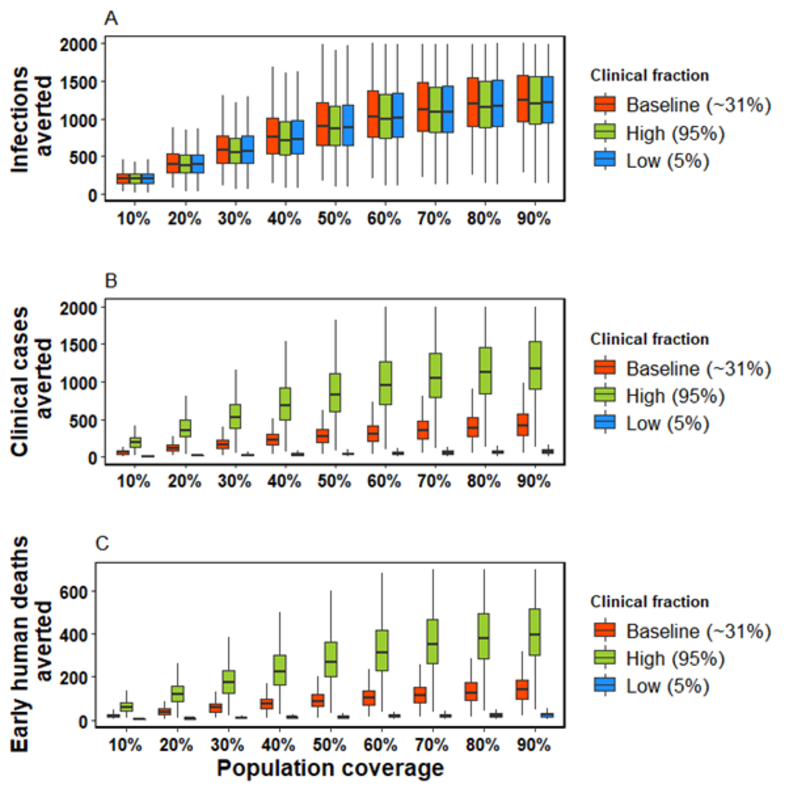

Supplement: S6 Fig — Increase in vaccination coverage among humans results as expected in increased epidemiological impact, in infections (panel A), clinical cases (panel B) and deaths (panel C). We take extreme values for the clinical fraction parameter to show that a scenario where only 5% of infections are symptomatic (blue boxplots) results in a drastic decline in epidemiological impact in cases and deaths when compared against a highly symptomatic scenario (green boxplots). The baseline calibrated model is shown in orange, for which the mean clinical fraction is 31%. (TIF) [file pntd.0010454.s006.tif]
