# Supplementary material for: Transmission dynamics and vaccination strategies for Crimean-Congo haemorrhagic fever virus in Afghanistan: A modelling study
Source: PLoS Negl Trop Dis. 2022 May 23;16(5):e0010454. doi: 10.1371/journal.pntd.0010454 (PMC9166359; doi:10.1371/journal.pntd.0010454)
Supplement: S1 Text — (DOCX) [file pntd.0010454.s007.docx]

**S1 Text: Model Equations**

1. **Livestock model**

Transmission of CCHFV among livestock is described with a compartmental deterministic model with mathematical expressions written below (equations 1 to 6).

We initialise the model to reflect a baseline CCHFV prevalence in animals (i.e. detectable IgG titters against CCHFV – model compartment R) that resembles what has been reported in endemic countries. We use reports from Bulgaria for this purpose **Table A** [1]. The initial livestock population is set to be 15,193, according to local census as described in Table 1 in the main text. The simulation starts in April 2008 and ran at a monthly time-step until April 2019 to cover the period for which livestock and human data are available.

| Age Group (yrs) | CCHFV IgG Prevalence (%) |
| --- | --- |
| 0-1 | 29 |
| 1-2 | 48 |
| 2-3 | 80 |
| 3-4 | 87 |
| 4+ | 87 |

**Table A.** Age distributed CCHFV prevalence among livestock, as reported by Barthel et al [1]

Susceptible

$$\begin{matrix} \frac{dS_{a}\left( t \right)}{dt}=\left\{ \begin{matrix} b_{L}\left( 1-\frac{\sum_{a} \left( I_{a}\left( t \right)+R_{a}\left( t \right) \right)}{N_{L}\left( t \right)} \right)+Ri\left( t \right)D_{aL}^{-1}+R_{a}\left( t \right)D_{mL}^{-1}-S_{a}\left( t \right)\left( \lambda_{L}\left( t \right)+\kappa v\left( t \right)+\mu_{a} \right)+\sum_{i=1}^{5} Z_{i,a}S_{i}\delta& for a=1 \\ R_{a}\left( t \right)\left( D_{mL} | -1 \right)-S_{a}\left( t \right)\left( \lambda L\left( t \right)+\left( \kappa\right)v\left( t \right)+\mu_{a} \right)+\sum_{i=1}^{5} Z_{i,a}S_{i}\delta& fora>1 \end{matrix} \right. \end{matrix}$$

*(Eq.1)*

Transient colostrum immunity

$$\frac{dRi\left( t \right)}{dt}=b_{L}\left( \frac{\sum_{a} I_{a}\left( t \right)+R_{a}\left( t \right)}{N_{L}\left( t \right)} \right)-Ri\left( t \right)D_{aL}^{-1}-Ri\left( t \right)\mu_{a}$$

*(Eq.2)*

Infectious livestock

$$\frac{dI_{a}\left( t \right)}{dt}=S_{a}\left( t \right)\lambda_{L}\left( t \right)-I_{a}\left( t \right)\left( {D_{iL}}^{-1}+\mu_{a} \right)+\sum_{i=1}^{5} Z_{i,a}I_{i}\delta$$

*(Eq.3)*

Recovered

$$\frac{dR_{a}\left( t \right)}{dt}=I_{a}\left( t \right)\left( {D_{iL}}^{-1} \right)-R_{a}\left( t \right)\left( {D_{mL}}^{-1}+\mu_{a} \right)+\sum_{i=1}^{5} Z_{i,a}R_{i}\delta$$

*(Eq.4)*

Newly vaccinated

$$\frac{dV_{a}\left( t \right)}{dt}=S_{a}\left( t \right)\kappa v\left( t \right)-V_{a}\left( t \right)\left( {D_{pV}}^{-1}+\mu_{a} \right)+\sum_{i=1}^{5} Z_{i,a}V_{i}\delta$$

*(Eq.5)*

Vaccine immunity

$$\frac{dP_{a}\left( t \right)}{dt}=V_{a}\left( t \right){D_{pV}}^{-1}-P_{a}\left( t \right)\left( \mu_{a} \right)+\sum_{i=1}^{5} Z_{i,a}P_{i}\delta$$

*(Eq.6)*

Age transition identity matrix

$Z_{i,j}=\left[ \begin{matrix} -1 & 0 & 0 & 0 & 0 \\ 1 & -1 & 0 & 0 & 0 \\ 0 & 1 & -1 & 0 & 0 \\ 0 & 0 & 1 & -1 & 0 \\ 0 & 0 & 0 & 1 & 0 \end{matrix} \right]$ *,for j =1,…,5, and i=1…5*

*(Eq.7)*

Force of infection in Livestock

$\lambda_{L}=\beta_{L}\frac{\sum_{a} I_{a}}{N_{L}}$

*(Eq.8)*

With environmental-driver-dependent transmission probability per-capita

$$\beta_{L}=\frac{R_{L}\left( t \right)}{D_{iL}}$$

*(Eq.9)*

Where *R_L_(t)* is the reproduction number at each point in time *t.*

*R_L_(t)* is defined according to the environmental driver used in the model. In section 3 of this document we describe the conditions set for each driver. As mentioned in the main text, we use environmental drivers as a proxy for tick-activity. Hence, we incorporate to our best knowledge, how this drivers might affect such activity.

1. **Human spillover model**

CCHFV transmission into humans in the model occurs as a function of the prevalence of CCHFV in livestock and a calibrated risk factor for farmers and non-farmers which implies the intensity of contact with animals and also the differential excess risk in farmers relative to other occupations. Here we assume that transmission into humans occurs only as a result of contact with animals and not with infectious humans. Contact with animals cover at least two potential routes: contact with animal fluids, and tick bites from ticks feeding in infectious livestock.

This spillover event is likely to be subject to stochastic variations, therefore we write a spillover model for humans as a discrete compartmental stochastic model that follows a SEIRS structure (see Fig 1 in main text and S2 Fig). Model states and transitions described below.

We initialise the model to reflect a baseline CCHFV prevalence in of ~9.5% among farmers and ~4% in other occupations. Here we assume that baseline prevalence is ~30% less than the data used for target during calibration. The initial human population and occupation distribution is detailed in Table 1 in the main text. The simulation starts in April 2008 and ran at a monthly time-step until April 2019 to cover the period for which livestock and human data are available.

Susceptible humans

$$\hat{S_{k}}\left( t+1 \right)=\hat{S_{k}}\left( t \right)+Binomial\left[ b_{H},\hat{N_{k}}\left( t \right) \right]+Binomial\left[ \left( {D_{mH}}^{-1} \right),\hat{R_{k}}\left( t \right) \right]-Binomial\left[ \lambda_{k}\left( t \right),\hat{S_{k}}\left( t \right) \right]-Binomial\left[ \kappa v\left( t \right),\hat{S_{k}}\left( t \right) \right]-Binomial\left[ \mu_{H},\hat{S_{k}}\left( t \right) \right]$$

*(Eq.10)*

Exposed humans

$$\hat{E_{k}}\left( t+1 \right)=\hat{E_{k}}\left( t \right)+Binomial\left[ \lambda_{k}\left( t \right),\hat{S_{k}}\left( t \right) \right]-Binomial\left[ {{D_{lH}}^{-1}}^{-1},\hat{E_{k}}\left( t \right) \right]-Binomial\left[ \mu_{H},\hat{E_{k}}\left( t \right) \right]$$

*(Eq.11)*

Infectious humans

$$\hat{I_{k}}\left( t+1 \right)=\hat{I_{k}}\left( t \right)+Binomial\left[ {{D_{lH}}^{-1}}^{-1},\hat{E_{k}}\left( t \right) \right]-Binomial\left[ \frac{{D_{iH}}^{-1}}{\left( \mu_{iH}+{D_{iH}}^{-1} \right)},totalEvents_{k}\left( t \right) \right]-Binomial\left[ \frac{\mu_{iH}}{\left( \mu_{iH}+{D_{iH}}^{-1} \right)},totalEvents_{k}\left( t \right) \right]-Binomial\left[ \mu_{H},\hat{I_{k}}\left( t \right) \right]$$

*(Eq.12)*

Recovered humans

$$\hat{R_{k}}\left( t+1 \right)=\hat{R_{k}}\left( t \right)+Binomial\left[ \frac{{D_{iH}}^{-1}}{\left( \mu_{iH}+{D_{iH}}^{-1} \right)},totalEvents_{k}\left( t \right) \right]-Binomial\left[ \left( {D_{mH}}^{-1} \right),\hat{R_{k}}\left( t \right) \right]-Binomial\left[ \mu_{H},\hat{R_{k}}\left( t \right) \right]$$

*(Eq.13)*

Newly vaccinated humans

$$\hat{V_{k}}\left( t+1 \right)=\hat{V_{k}}\left( t \right)+Binomial\left[ \kappa v\left( t \right),\hat{S_{k}}\left( t \right) \right]-Binomial\left[ {D_{pV}}^{-1},\hat{V_{k}}\left( t \right) \right]-Binomial\left[ \mu_{H},\hat{V_{k}}\left( t \right) \right]$$

*(Eq.14)*

Newly vaccine protected humans

$$\hat{P_{k}}\left( t+1 \right)=\hat{P_{k}}\left( t \right)+Binomial\left[ {D_{pV}}^{-1},\hat{V_{k}}\left( t \right) \right]-Binomial\left[ \mu_{H},\hat{P_{k}}\left( t \right) \right]$$

*(Eq.15)*

Force of infection in humans

Force of infections in human is here a function of the prevalence of infectious livestock at time *t* and a transmission probability $\beta_{F}$. For other occupations we include a factor O that is estimated to reflect the risk ration between farmers and other human groups.

$$\lambda_{k}\left( t \right)=\left\{ \begin{aligned} \begin{matrix} \beta_{F}\frac{\sum_{a} I_{a}\left( t \right)}{N_{L}\left( t \right)} & ,for farmers\left( k=1 \right) \end{matrix} \\ \begin{matrix} O\beta_{F}\frac{\sum_{a} I_{a}\left( t \right)}{N_{L}\left( t \right)},for others\left( k=2 \right) \end{matrix} \end{aligned} \right.$$

*(Eq.16)*

To estimate new fatalities and recoveries we use the competing hazard formula, relying on the known CFR for CCHFV(see **Table 1** in main text). We write the corresponding competing mortality hazard as follows:

$$\mu_{iH}=\frac{{CFR}_{cchfv}\left( {D_{iH}}^{-1} \right)}{1-{CFR}_{cchfv}}$$

*(Eq.17)*

Then we calculate the total number of events (new fatalities and recoveries) occurring at time *t* in the infectious compartment:

$$totalEvents_{k}\left( t \right)=Binomial\left[ \left( {D_{iH}}^{-1}+\mu_{iH} \right),\hat{I_{k}}\left( t \right) \right]$$

*(Eq.18)*

Finally, we split the number of events into new recoveries and new fatalities as seen in equations 13-14.

1. **Environmental dependant reproduction number in livestock**

In this section we present how each environmental driver is accounted for in the model. Data sourcing and processing of environmental driver variables are available in section 4 of this document.

Temperature-dependent reproduction number in livestock

Temperature is a known driver of viral transmission as it is a main factor driving *Hyalomma spp*. Adult *Hyalomma spp*. activity is known to occur above 12°C[2,3] and increases as temperature increases. Once temperature reaches above 30°C, ticks prefer to bury into soil[4]. The force of infection amongst livestock *λL(t)* (*Eq.8,9*) was therefore modelled as a linear function of temperature from 12 to 30^O^ and linearly declining force of infection at temperatures above 30^O^.

$$R_{L}\left( t \right)=\left\{ \begin{matrix} 0 & ,ifT<12^{\circ}C \\ A\left( T\left( t \right)-T_{min} \right) & ,if12^{\circ}C\leq T\leq30^{\circ}C \\ A\left( 30-\left( T\left( t \right)-30 \right)-T_{min} \right) & ,ifT>30^{\circ}C \end{matrix} \right.$$

*(Eq.19)*

Where *A* is the temperature dependent transmission factor, and *T(t)* is soil temperature in $^{\circ}C$ for Herat at time *t* of run-time (from ERA5 data).

Saturation-deficit-dependent reproduction number in livestock

Saturation deficit, a measure of the drying capacity of the air, is an implicit measure of air temperature. In our exploration of different environmental drivers for explaining tick activity cycles in Herat, we test the performance of saturation deficit as an indicator. To keep tick activity within the temperature-define range (12 -30 degrees C), as explained above for the temperature dependent reproduction number, we fit a polynomial function in order to predict the saturation deficit values that correspond to specific soil temperature levels (**Fig A**). This allows us to use the temperature dependant function while using saturation deficit trends.


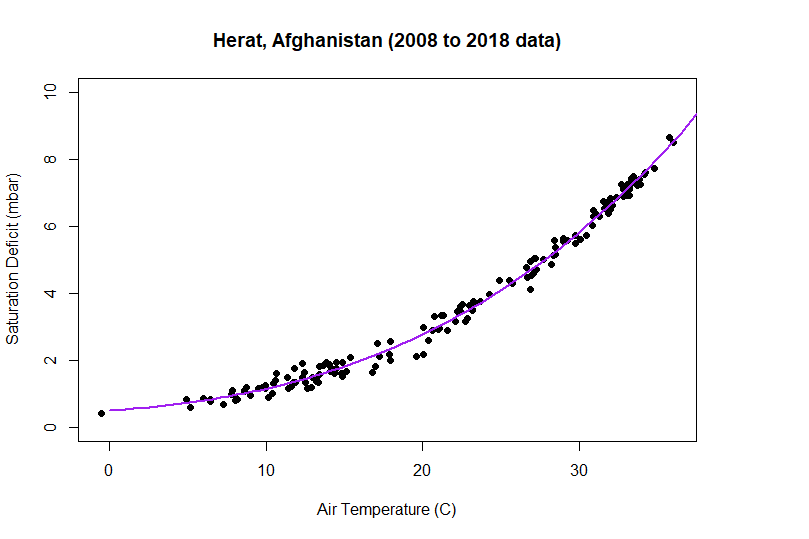


**Fig A.** Polynomial model prediction model of Saturation deficit on Air temperature. Black dots show the corresponding coordinates of Air temperature/ Saturation deficit for specific dates (months between April 2008 to April 2019) in Herat, Afghanistan. The purple line shows predicted values from a polynomial model fit.

Therefore, we describe the saturation deficit dependent reproduction number as follows:

$$R_{L}\left( t \right)=\left\{ \begin{matrix} 0 & ,ifsatdef<6.415mbar \\ A\left[ min\left( satdef,26.79mbar \right)-min\left( satdef \right) \right] & ,if26.79mbar\geq satdef\geq6.415mbar \\ A\left[ 26.79mbar-\left( satdef-26.79mbar \right)-min\left( satdef \right) \right] & ,ifsatdef>26.79mbar \end{matrix} \right.$$

*(Eq.20)*

Where *A* is here the saturation deficit dependent transmission factor, and *satdef(t)* is saturation deficit for Herat at time *t* of run-time (from ERA5 data).

Reproduction number in livestock for other environmental drivers

For the non-temperature drivers we define a force of infection as a linear function of the driver and the calibrated factor A. We don’t impose limitations as caps or thresholds for these other drivers, namely, relative humidity and Normalized Difference Vegetation Index (NDVI). R_L_(t) is then defined as follows:

$$R_{L}\left( t \right)=A\left[ driver\left( t \right)-min\left( driver \right) \right]$$

*(Eq.21)*

Where *driver* is to reflect the environmental factor used, either NDVI or relative humidity.

**References**

1. Barthel R, Mohareb E, Younan R, Gladnishka T, Kalvatchev N, Moemen A, et al. Seroprevalance of Crimean–Congo haemorrhagic fever in Bulgarian livestock. Taylor & Francis. 2014;28: 540–542. doi:10.1080/13102818.2014.931685

2. Hyalomma marginatum - Factsheet for experts. [cited 22 Dec 2021]. Available: https://www.ecdc.europa.eu/en/disease-vectors/facts/tick-factsheets/hyalomma-marginatum

3. Estrada-Peña A, Vatansever Z, Gargili A, Ergönul Ö. The trend towards habitat fragmentation is the key factor driving the spread of Crimean-Congo haemorrhagic fever. Epidemiol Infect. 2010;138: 1194–1203. doi:10.1017/S0950268809991026

4. Valcárcel F, González J, González MG, Sánchez M, María Tercero J, Elhachimi L, et al. insects Comparative Ecology of Hyalomma lusitanicum and Hyalomma marginatum Koch, 1844 (Acarina: Ixodidae). doi:10.3390/insects11050303
