# Supplementary material for: Transmission dynamics and vaccination strategies for Crimean-Congo haemorrhagic fever virus in Afghanistan: A modelling study
Source: PLoS Negl Trop Dis. 2022 May 23;16(5):e0010454. doi: 10.1371/journal.pntd.0010454 (PMC9166359; doi:10.1371/journal.pntd.0010454)
Supplement: S2 Text — (DOCX) [file pntd.0010454.s008.docx]

**S2 Text: Livestock demographic model**

The livestock model as explained above, is stratified into 5 age groups reflecting one year range for each group, except the last age group includes all those aged 4+ years. In order to obtain an equilibrium demographic model for livestock we simultaneously estimated the mortality rates for each age group. For this we constructed an age stratified births and deaths model, and optimised mortality rates for each age group to match a known livestock age distribution from a survey in Mauritania[1] using a simple optimisation algorithm in R (*bmle library*). In the absence of Afghani data on livestock age structure we choose data from Mauritania, a country with extensive livestock production for which this information has been published. We adapt the age distribution in livestock from Schulz et al [1], to reflect a mean life expectancy of 5 years. The total livestock population was obtained from FAO’s 2003 survey[2]. **Fig A** shows the resulting age distribution.


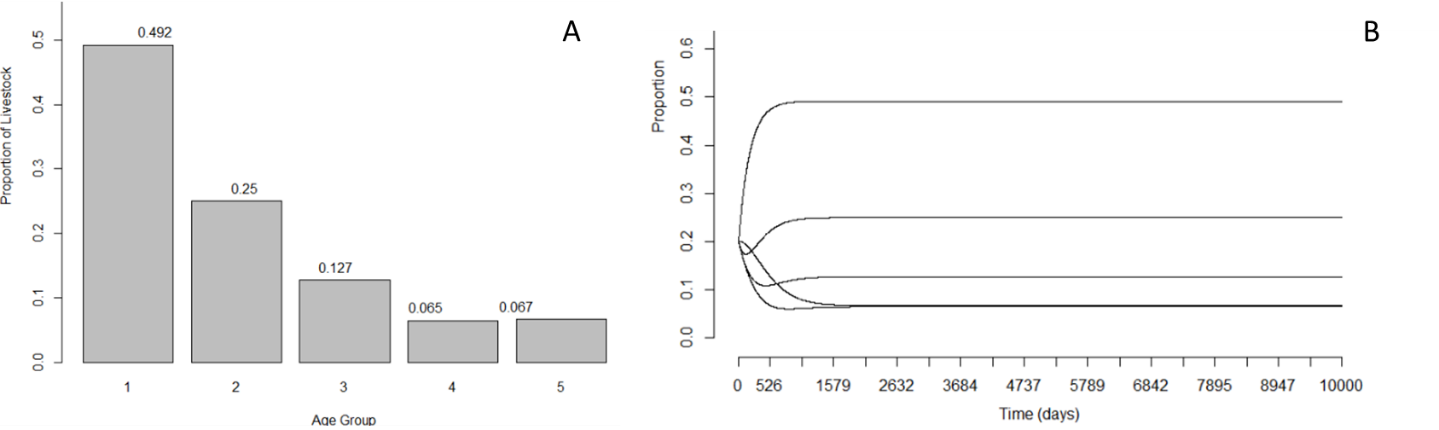


**Fig A**. In panel A, a final age distribution in cattle into 5 age yearly age groups. In panel B, the time trend of age group proportions as they reach equilibrium.

**References**

1. Schulz A, Barry Y, Stoek F, Ba A, Schulz J, Haki ML, et al. Crimean-congo hemorrhagic fever virus antibody prevalence in mauritanian livestock (Cattle, goats, sheep and camels) is stratified by the animal’s age. PLoS Neglected Tropical Diseases. 2021;15. doi:10.1371/JOURNAL.PNTD.0009228

2. FAO. Afghanistan national livestock census 2002-2003. Rome; 2008.
