# Supplementary material for: Transmission dynamics and vaccination strategies for Crimean-Congo haemorrhagic fever virus in Afghanistan: A modelling study
Source: PLoS Negl Trop Dis. 2022 May 23;16(5):e0010454. doi: 10.1371/journal.pntd.0010454 (PMC9166359; doi:10.1371/journal.pntd.0010454)
Supplement: S3 Text — (DOCX) [file pntd.0010454.s009.docx]

**S3 Text: Model calibration**

We denote by *θ* the vector of input parameters, for all model inputs subject to uncertainty. For a given parameter set *θ*, we followed the steps:

1. Run an instance of the Livestock model
2. Record Livestock output including a vector of the prevalence of infectious livestock at each time point.
3. Run an instance of the human spillover model with the relevant *θ* and the vector of prevalence in livestock (to constrict force of infection, see Eq. 19-20 in **S1 Text**)
4. Record human spillover model output
5. Calculate global posterior for the model and calibration targets described in **Table A**.

To compare these model projections with data *D*, we defined the posterior density *π(θ)* as:

$\pi\left( \theta\right)\propto L\left( D│\theta\right).P\left( \theta\right)$ *(Eq.26)*

Where *L* is the likelihood of the data *D* given models with parameters *θ* and *P* is the joint prior distribution for *θ*. For *P*, we took independent uniform distributions over the ranges shown in **Table 1** in the main text. The likelihood *L* was constructed as follows. For count outputs (i.e., cases, fatalities) we estimate the *Poisson* likelihood, and the binomial likelihood for binary outcomes like prevalence. In particular, we determined the mean and variance of these distributions in order for the 2.5th, 50th and 97.5th percentiles to match respectively the lower, mid and upper ranges of estimates. For a given parameter set θ, we then constructed the overall likelihood π(θ) as a product of these distributions over all calibration targets listed in **Table A**. In practice we computed the logarithm of π(θ), thus taking the sum of the logarithms of each of the probability densities involved.

With *π(θ)* thus defined, we sampled the posterior density using a Markov Chain Monte Carlo approach. In brief, this approach implements a random walk through the space of parameter values *θ* to obtain an unbiased sample of the posterior density. We implemented the Metropolis Hastings algorithm with adaptive gaussian proposal existing in the package “fitR” for R [1]. For the set of parameter values thus obtained, we took every tenth element to reduce autocorrelation, thus yielding an ‘ensemble’ of parameters θ_1_,θ_2_,… ; This ensemble captures simultaneously the uncertainty in the parameter inputs, as well as in the calibration data. Then, to estimate uncertainty in a given simulated output φ (e.g. in the reduction of incidence with a given coverage of intervention), we simulated this output *φ_i_* for every *θ_i_*. We finally estimated uncertainty in *φ_i_* by determining its 2.5th, 50th and 97.5th percentiles. For graphic depiction of model fits to data see **Fig 2** in the main text 5. **Fig A** shows the MCMC diagnostic outputs for each parameters, and **Fig B** shows density plots for the calibrated parameters for the Saturation deficit model.

Convergence was assessed visually by inspecting the trace plots of the calibrated parameters and also through estimation of the Gelman –Rubin convergence diagnostic [2], computed as follows:

$R=\frac{v}{w}$, *(Eq.27)*

Where $v$ is the posterior variance estimate of the combined chains and *w* is the within-chain variance. If the chains have converged to the target posterior distribution, then $R$ (also known as the **potential** **scale reduction factor**) should be close to 1. As a rule of thumb, values below 1.1 are typically considered to indicate convergence[2]. **Fig C**, shows the estimate of the Potential scale reduction factor (PSRF) for the six calibrated parameters.

| **Calibration target** | **Description** | **Year(s)** | **Source** |
| --- | --- | --- | --- |
| Livestock seroprevalence of CCHFV | Age stratified IgG seroprevalence from a serosurvey in Herat (n=132) | 2009 | Mustafa *et al*. 2011[3] |
| Human seroprevalence of CCHFV | IgG seroprevalence I humans by occupation in Herat (n=330) | 2009 | Mustafa *et al*. 2011[3] |
| Monthly Human CCHFV cases reported | Reported human cases in Herat. Cases in 2018 at national level and assumed that ~62% are from Herat according to Niazi et al.[4] | 2008, 2017, 2018 | Mofleh et al[5]  Niazi et al[4]  Sahak et al[6] |
| Yearly Human CCHFV cases reported | Yearly aggregated cases reported nationally. Assumed that ~62% are from Herat according to Niazi et al.[4] | 2009, 2010, 2010, 2011, 2012, 2013, 2014, 2015, 2016 | Niazi et al[4]  Sahak et al[6] |
| Yearly Human CCHFV fatalities reported | Yearly aggregated deaths reported nationally. Assumed that ~62% are from Herat according to Niazi et al.[4] | 2009, 2010, 2010, 2011, 2012, 2013, 2014, 2015, 2016 | Niazi et al[4]  Sahak et al[6] |

**Table A: Calibration target datasets for CCHFV in Herat, Afghanistan**

**
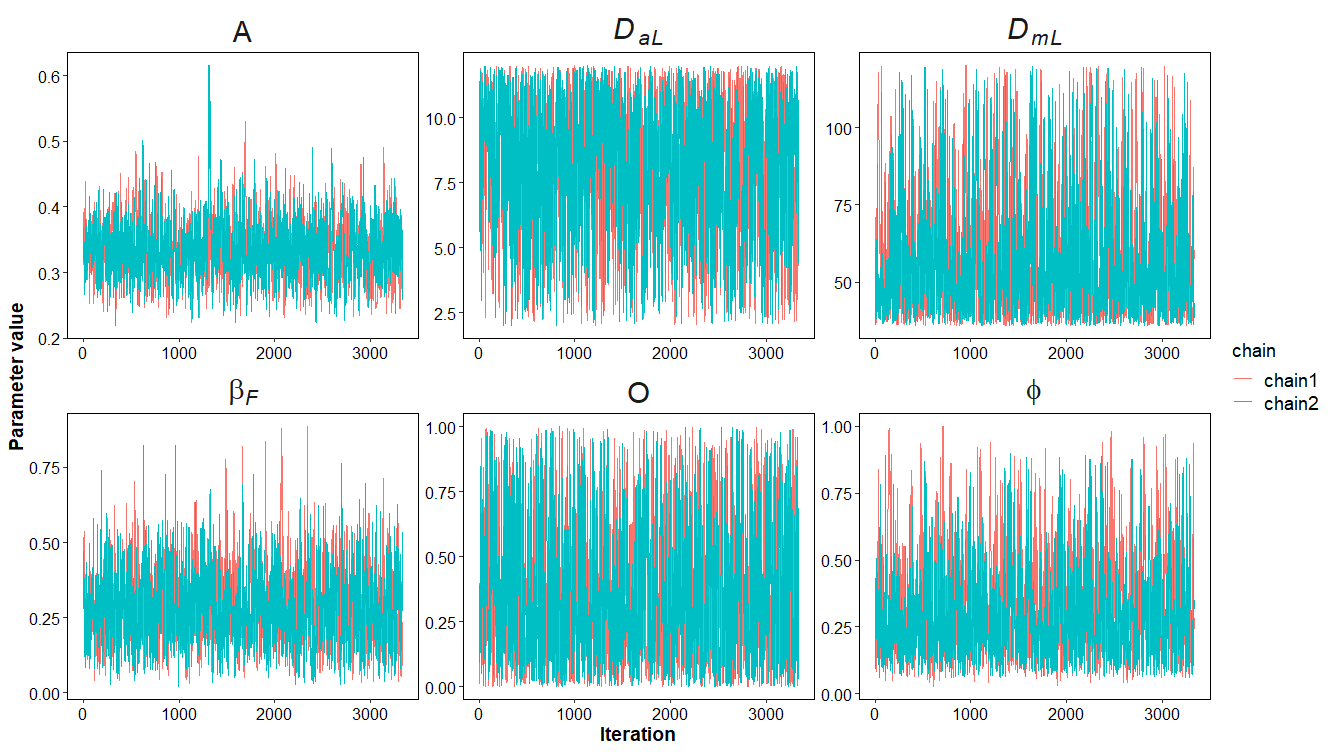
**

**Fig A: MCMC Trace plots** for the 6 calibrated parameters. Plots show two 100K chains (after burn and thinning). These results reflect calibration for the final selected model (saturation deficit). Trace plots show a good mixing of the chains which suggests convergence to a stationary distribution.


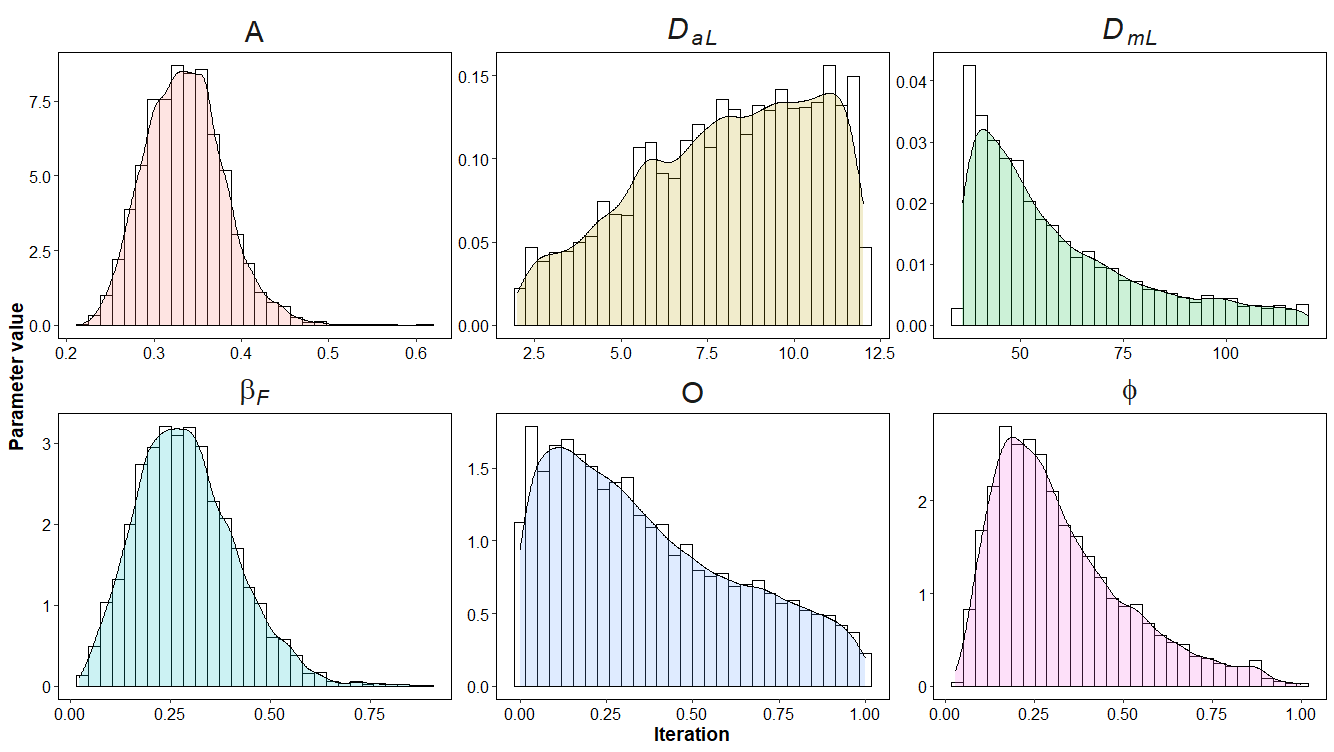


**Fig B. Density plots.** Histograms and density plots sampled from the posterior distribution obtained through MCMC. Each parameter reflects the combined, burned, thinned and resampled posterior for the calibrated parameters. These are results for the final selected model, i.e., with saturation deficit as the environmental driver.


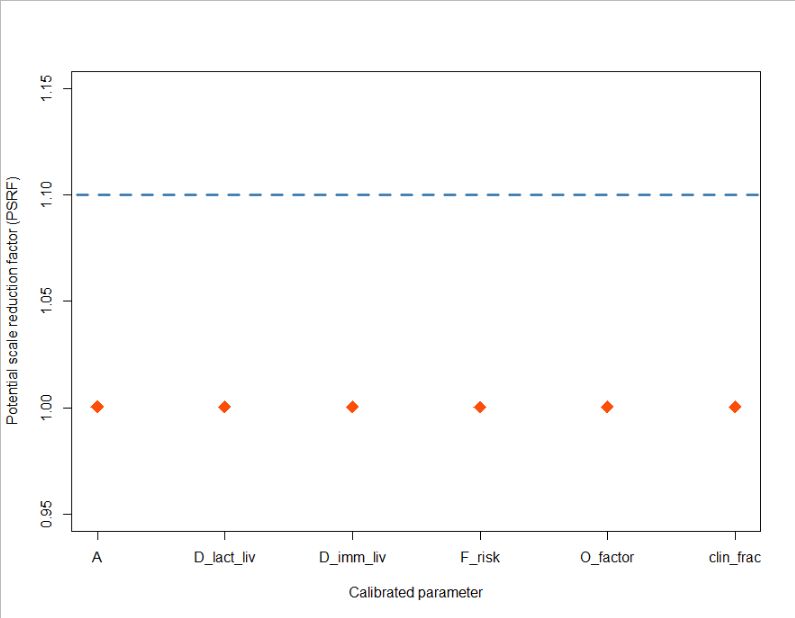


**Fig C: Gelman-Rubin diagnostic** for the six calibrated parameters. Orange dots show the point estimate of the Potential Scale Reduction Factor for each calibrated parameter, and the dashed blue line shows the rule-of-thumb convergence threshold. This suggests good convergence for all parameters. These are results for the final selected model, i.e., “saturation deficit driver” model. Variations in specific PSRF are not evident here given the scale of the plot.

**References**

1. Title Tool box for fitting dynamic infectious disease models to time series. 2019.

2. Gelman A, Rubin DB. Inference from Iterative Simulation Using Multiple Sequences. Statistical Science. 1992;7: 457–472. doi:10.1214/ss/1177011136

3. Mustafa ML, Ayazi E, Mohareb E, Yingst S, Zayed A, Rossi CA, et al. Crimean-Congo Hemorrhagic Fever, Afghanistan, 2009. Emerging Infectious Diseases. 2011;17: 1940. doi:10.3201/EID1710.110061

4. Niazi A, Jawad M, Amirnajad A, Durr P, Williams D. Crimean-Congo Hemorrhagic Fever, Herat Province, Afghanistan, 2017. Emerg Infect Dis. 2019;25: 1596–1598. doi:10.3201/EID2508.181491

5. Mofleh J, Ahmad Z. Crimean-Congo haemorrhagic fever outbreak investigation in the Western Region of Afghanistan in 2008. Eastern Mediterranean health journal = La revue de sante de la Mediterranee orientale = al-Majallah al-sihhiyah li-sharq al-mutawassit. 2012;18: 522–526. doi:10.26719/2012.18.5.522

6. Sahak M, Arifi F, Saeedzai S. Descriptive epidemiology of Crimean-Congo Hemorrhagic Fever (CCHF) in Afghanistan: Reported cases to National Surveillance System, 2016-2018. Int J Infect Dis. 2019;88: 135–140. doi:10.1016/J.IJID.2019.08.016
