# Supplementary material for: Transmission dynamics and vaccination strategies for Crimean-Congo haemorrhagic fever virus in Afghanistan: A modelling study
Source: PLoS Negl Trop Dis. 2022 May 23;16(5):e0010454. doi: 10.1371/journal.pntd.0010454 (PMC9166359; doi:10.1371/journal.pntd.0010454)
Supplement: S4 Text — (DOCX) [file pntd.0010454.s010.docx]

**S4 Text: Environmental drivers**

In the absence of tick activity data we use (and test) environmental drivers as a surrogate measure of tick activity. It has been observed that *Hyalomma spp*. similarly to other tick species, are strongly driven in their reproduction cycles and also feeding activity by different climatic and land composition variables[1,2]. Some of these variables are easily summarised by atmospheric indicators (soil temperature, relative humidity) and others require some calculation or a more complex approach (Saturation deficit , NDVI).

**Environmental data and indicator construction**

Atmospheric data was retrieved for Herat, Afghanistan, ERA5 climate data reanalysis, and accessed through Copernicus project interface [3,4]. This dataset contains time series of multiple climatic variables around the planet.

We set up a polygon around Herat with Lat/Long coordinates (35°Lat / 62.1°Long - 34°Lat /62.5°Long).

For a description of drivers and its application in the model refer to **Table 2** in the main text.

1. Soil temperature

Retrieved for the selected area from April 2008 to Jan 2019. The time series of average monthly soil temperature was use directly as input into the model.

1. Relative Humidity

Relative humidity (RH) is not directly collected by the ERA5 project, but can be estimated using readily available variables like the 2m air temperature (airT2m) and the dewpoint temperature(dewpoint).

$$RH=\frac{actualvapourpressure}{saturatedvapourpressure}$$

*(Eq.22)*

$$actualvapourpressure=\frac{exp\left( 17.625\left( dewpointT \right) \right)}{\left( 243.04+dewpointT \right)}$$

*(Eq.23)*

$$saturatedvapourpressure=\frac{exp\left( 17.625\left( airT2m \right) \right)}{\left( 243.04+airT2m \right)}$$

*(Eq.24)*

1. Saturation deficit

Saturation deficit (SD) is not directly collected by the ERA5 project, but can be constructed from relative humidity (RH) and the ERA5 2m air temperature (airT2m)[5]

$$saturationdeficit=\left( 1-RH \right)\left( 4.9463 \right)exp\left( 0.0621\left( airT2m \right) \right)$$

*(Eq.25)*

1. Normalized Difference Vegetation Index (NDVI)

NDVI is an index for the density of vegetation in a specific area. It is calculated by assessing the reflectance of vegetation as estimated with satellite images. NASA’s Earth-Data project collates this information and estimates time series of NDVI, which we used and gathered from the MODIs subsets[6]. We use the same polygon of area around Herat described above. The time series data of monthly NDVI measures for the area is used directly into the model.

**References**

1. Estrada-Peña A, Jameson L, Medlock J, Vatansever Z, Tishkova F. Unraveling the ecological complexities of tick-associated crimean-congo hemorrhagic fever virus transmission: A gap analysis for the western palearctic. Vector-Borne and Zoonotic Diseases. 2012;12: 743–752. doi:10.1089/VBZ.2011.0767

2. Estrada-Peña A, Vatansever Z, Gargili A, Ergönul Ö. The trend towards habitat fragmentation is the key factor driving the spread of Crimean-Congo haemorrhagic fever. Epidemiol Infect. 2010;138: 1194–1203. doi:10.1017/S0950268809991026

3. Climate reanalysis | ECMWF. [cited 22 Dec 2021]. Available: https://www.ecmwf.int/en/research/climate-reanalysis

4. Climate reanalysis | Copernicus. [cited 22 Dec 2021]. Available: https://climate.copernicus.eu/climate-reanalysis

5. Bregnard C, Rais O, Voordouw MJ. Climate and tree seed production predict the abundance of the European Lyme disease vector over a 15-year period. Parasit Vectors. 2020;13. doi:10.1186/S13071-020-04291-Z

6. MODIS Subsets | Earthdata. [cited 22 Dec 2021]. Available: https://earthdata.nasa.gov/earth-observation-data/near-real-time/rapid-response/modis-subsets
