# Supplementary material for: Transmission dynamics and vaccination strategies for Crimean-Congo haemorrhagic fever virus in Afghanistan: A modelling study
Source: PLoS Negl Trop Dis. 2022 May 23;16(5):e0010454. doi: 10.1371/journal.pntd.0010454 (PMC9166359; doi:10.1371/journal.pntd.0010454)
Supplement: Attachment — Submitted filename: Reviewers_comments_final.docx [file pntd.0010454.s011.docx]

**Responses to Reviewer comments**

We thank the reviewers for their extremely careful and constructive reading of our manuscript. We have made several additions to address their comments: we hope the reviewers will agree that these changes have substantially improved the manuscript. Changes are shown ‘tracked’ in the manuscript and supporting information, and below we provide point-by-point responses to each comment (in blue).

**Reviewer #1:**

1) There were a number of inputs and values that were “estimated” and it was not always clear how these inputs and values were determined.

*We added further clarification at the footnote of parameters table 1 in the main text (see p. 10), to note that estimated values reflect a sample from the posterior distribution resulting from the model calibration procedure*

2) It was not clear if the authors accounted for varying contact rates with livestock between farmers and the general population. The calibrated model shows the proportion of contact for other occupational groups was 0.3 and transmission from livestock to farmers was 0.28. Was this assumption based on farmers having higher pre-existing CCHF immunity?

*We account for variations in transmission coefficient (β_F_) among farmers and in the transmission factor (O) for other occupations relative to farmers. The term β_F_ here reflects the product of the average contact rate and the per-contact transmission probability. Therefore, we do not explicitly model variable contact patterns between humans and livestock. We have added a better description of these terms in parameters table 1 in main text (see p.10)*

3) The authors estimate waning CCHF immunity on livestock after 5 years. What was this based on? And even if waning immunity, by what factor would this reduce protection against re-infection.

*It is widely assumed that CCHF provides lifelong immunity in livestock. For this modelling study we take an agnostic approach towards this assumption, partly based on the fact that there is no definitive evidence to support lifelong protection after infection. For this reason we relaxed this assumption to allow a waning period of 5 years on average. By making this assumption we obtain the best model fit to age stratified prevalence data. We add* ***figure S9*** *in the supporting information, showing the model calibration when we assume lifelong immunity, and we present the DIC model results for this scenario in new* ***figure S10*** *(page 18 in supporting information).*

4) The model assumes these animals after 5 years are able to be re-infected, but the general consensus for CCHF is infection confers lifelong immunity for both animals and humans.

*See response to point 3 above*

5) The human immunity is also estimated at 10 years, and we presume lifelong immunity, or at least protective immunity even if waning measurable antibody titters.

*We assume that immunity (protection as well as antibody titres), as this is the model structure that best reproduces the known data for Herat. In the absence on more definitive evidence about lifelong protection in humans we believe this assumption is plausible.*

6) The authors estimate the fraction of human CCHF infections that result in clinical cases was .31. Based on first-hand experience and study this seems very low and could be an underestimate.

*We agree with the reviewer. The studies on symptomatic fraction available are susceptible to several limitations. This uncertainty was the main motivation for including this parameter in the calibration procedure. To recognise this point and highlight the need of new studies on this matter we have added a discussion point (see p.20 lines 279 to 283 in the main text).*

7) These assumptions may impact the model outputs if not validated or evidence is available for documented re-infection of humans and/or animals

*We agree. See the point above. See also reply to comment 11*

8) Authors state ticks bury in soil once temp goes beyond 30 degrees. What is the shape of the function for declining transmission? Is it linear? Exponential? What assumptions are made? Was this determined by model calibration?

*We use a capped linear function to reflect these limits with regards tick activity relative to temperature. The process is detailed in section 2.3 in the supporting information (page 8). We added a reference to this description in the main text (see page7, line 127)*

9) The first point of discussion highlights the lack of tick vector data in which to calibrate and use for the model. It is not stated in the manuscript what the assumption of percent of total ticks are infected with CCHF and how this impacts the potential infectivity of cattle/human populations. From practical experience tick infectivity could be low, but the documented seropositivity in livestock is often very high. Therefore the estimate for livestock-livestock CCHF transmission could be happening at a higher rate than estimated in the model, although the estimates used could offset each other and therefore not have any impact on this model or effect any of the resulting outputs.

*In the absence of tick activity data we model transmission between livestock using a proxy function that reflects tick activity as a function of time-varying environmental drivers. One of the benefits of this approach is that assumptions about tick infectivity do not have to be made explicitly. Instead, we use the climatic indicator, the prevalence of CCHFV in livestock and a scaling factor for the environmental driver, to construct a force of infection between livestock. This is explained in the Methods section (mathematical model subsection). However, we added further clarification to the caption of Fig 1-page 7).*

10) The authors state again that an estimated 31% of spillover cases become symptomatic and therefore detectible by surveillance. As stated above, this seems too low of an estimate. The two references (29, 30) showing that the potential for asymptomatic infection for CCHF could be higher. I believe the authors may have misinterpreted these references and greatly overestimated the number of potential asymptomatic infections. One of the references use a serological assay known for its overestimation of the true IgG seropositivity. The second reference is a validation study of IgG positive CCHF cases from three independent serosurveys where the IgG seropositivity that could be attributable to asymptomatic CCHF as less than 5%.

*See response to point 6 above on this same subject (see p.20 lines 279 to 283).*

11) Generally, the effects of vaccination on the targeted populations and the resulting reduction in estimated CCHF human cases is as would be expected given the analysis. The only change to the results could be the absolute number calculated from the model if some of the assumptions were to be adjusted up or down based on more real-world estimates.

*This is indeed a very important point, and to show how sensitive our estimations of epidemiological impact are relative to clinical fraction, we have added figure S11 in supporting information (see page 19), and added a reference to this plot in main text (see discussion, page 21 , lines 283-285).*

12) Overall, this manuscript is a good first analysis of the impact vaccination could have on human CCHF infections given the transmission dynamics in a specific geographic location in Afghanistan. It may not be as generalizable given the dynamics of CCHF varies so widely across different geographic locations and ecological niches. Some limitations the authors properly address, but lack of location specific variables/data points could bias their analysis if taken from locations distant from the study location (e.g CCHF data from Turkey) to help validate or calibrate the model.

*This an important point, and indeed a further exploration of other epidemiological settings for CCHF like Turkey and South Africa is currently underway and we aim to complete this analysis shortly. To highlight the importance of what we consider the natural progression of this work we have added to discussion in page 21, lines 306-307.*

**Reviewer #2:**

13) One research or one publication is not enough until some of the work is done in the different countries where CCHF is prevalent so as to give a comprehensive picture. The authors could edit their recommendations and conclusions accordingly.

*See reply to comment 12 above*

14) The manuscript could receive another read and make sure that the English drama is reading well especially in the abstract. For example I do not know the use of the word"course "as used in this abstract or are they meaning "Cases".

*The text has been proofread by all co-authors to satisfy readability and transparency in the use of English language. Regarding the use of “course” in the results section and abstract: we prefer the word “course” to doses, as it reflects the whole vaccination scheme which might comprise of one or more doses. In the specific mention in the abstract, it is intended to refer to the courses per case averted, this is the number of vaccinations delivered necessary to avert one clinical case. We have also changed the text on P16 (results) to clarify that we mean vaccine courses.*

**Reviewer #3:**

*Main text*

P 7: The formula at the end of Figure 1 caption should have N_L instead of N, like the equation on line 136.

*Has been corrected (see page 7)*

P 7: Lines 120—125 should continue the caption of Figure 1.

*Has been corrected (see page 7)*

P 10: What is the value of the monthly birth rate in livestock?

*The demographic model in livestock is described in section 3 of the supporting information (see page 10). As we mention, we bring the livestock model to a demographic equilibrium, by optimising age specific monthly mortality rates to achieve a target age distribution according to known data from elsewhere. Birth rates therefore are set to input the same number of animals dying each month. Mortality rates by age can be found in table 1 in the main text*

P 10: The notation for transmission from livestock to other occupational groups should be \beta_O.

*Has been added (see table 1 page 10)*

P 10: What is the value of the time to vaccine protection?

*Our assumption of 2 weeks to reach immunity has been corrected (see table 1 page 10)*

P 10: Lines 176–178 are an odd place for the ethics statement. Perhaps move it elsewhere.

*We have moved the ethical approval section to page 14 line 217*

P 17: On line 251, "panel B of Figure 3B" should be "Figure 3B" in parallel with "Figure 3D" on line 258.

*Has been corrected (see page 17 line 251)*

*Supplement*

P 4–6: What are the initial conditions for the livestock model? How long is period of time is simulated?

*We added this to the supporting information and model description. See pages 4-5 in the supporting information and table S1*

P 4: The second line of Eq. 1 is not rendered correctly.

*Equation 1 has been corrected )see page 5 supporting information)*

P 4: Comparing Eq. 2 to Figure S1, the term for births from recovered livestock (R_a(t)) is missing.
*See equation 2, page 2 in supporting information for corrected version*

P 5: In Eq. 3, the "L" following lambda should be a subscript.

*Corrected. See page 5 supporting information*

P 5: In Eq 7, for Z_{i,j}, the lower left term Z_{5,5} should be 0, not -1, as the animals 5th age group do not age out, only leave through mortality (mu_a).

*We are grateful to reviewers for spotting this error. It has been corrected to reflect the actual functioning of the model (see page 6 Eq 7 in supporting information)*

P 6–8: What is the time step used in the human model? What are the initial conditions for the human models? How long is period of time is simulated?

*We added initial conditions for clarification in page 6 of the supporting information*

P 8 & 9: I don't understand how saturation deficit, air temperature, and tick activity are related from the section and Figure S3. What are the black dots in the figure? Please explain further.

*We agree that this needed clarification. We added further explanations to page 9 and the caption of figure S3 in the supporting information.*

P 10: I found "with the last categories being those 4+ years" to be confusing. I recommend "except the last age group includes all those aged 4+ years" or similar.

*Corrected as suggested (see page 10 supporting information)*

P 10: "It has been observed that Hyalomma spp. similarly to other tick species, are strongly driven in their reproduction cycles and also feeding activity by different climatic and land composition variables." Please provide citations at the end of this sentence.

*Added references 3 & 7 to support this statement (see page 11 supporting information)*

P 11: "prevalent I" should be "prevalence".

*Corrected as suggested (see page 12 supporting information)*

P 12: "As a rule of thumb, values below 1.1 are typically considered to indicate convergence." Please provide a citation, perhaps to Gelman's book.

*Added reference 13 to Gelman & Rubin 1992 to support this statement (see page 13 supporting information)*

P 12: You refer to the test statistic (R with two dots) as the "scale reduction factor" then the "Potential scale reduction factor (PSRF)" in the same paragraph, with the latter also used in Figure S7. Please either use "potential" throughout or explain why "potential" is added later.

*This has been corrected for consistency to say Potential Scale Reduction Factor (see page 13 supporting information)*

P 12: "shoes" should be "shows".
*Has been corrected*

Yours sincerely,

**Dr Juan F Vesga (on behalf of all co-authors)**
